# A Scoping Review of Artificial Intelligence in Ocular Oncology

**DOI:** 10.3390/cancers18111698

**Published:** 2026-05-23

**Authors:** Vijitha S. Vempuluru, Swathi Kaliki

**Affiliations:** The Operation Eyesight Universal Institute for Eye Cancer, LV Prasad Eye Institute, Hyderabad 500034, Telangana, India; vijithavenus@gmail.com

**Keywords:** eye, tumor, cancer, ocular oncology, artificial intelligence, AI

## Abstract

**Objective**: To provide a comprehensive literature review of original work on artificial intelligence in ocular oncology. **Methods**: Scoping review of PubMed-indexed original articles (*n* = 94) on the use of artificial intelligence in ocular oncology, retrieved during the month of February 2026 and independently screened by two ocular oncologists. **Results**: Most of the literature on artificial intelligence (AI) in ocular oncology focuses on uveal melanoma and its differentials (*n* = 39, 41%), followed by retinoblastoma (*n* = 14, 15%) and orbital tumors (*n* = 12, 13%). The purpose of using the AI models was to screen, diagnose, and classify the disease (*n* = 59, 62%) and to treat, predict outcomes, and monitor the disease (*n* = 35, 37%). Most literature (*n* = 32, 34%) on AI in ocular oncology originates from China. Datasets comprised images in 78% (*n* = 73) of the studies, clinical parameters in 14% (*n* = 13), and omics data in 12% (*n* = 11). Most studies worked on developing AI models (*n* = 83, 88%), of which two reached a deployment stage. Few studies evaluated or incorporated pre-existing models (*n* = 11, 12%). Supervised learning strategy was most commonly employed (*n* = 75, 80%). Among studies that developed AI models, traditional machine learning architectures were used in 36, deep learning in 39, and a combination in 8. Most studies (*n* = 59, 63%) were at a Clinical AI Readiness Evaluator Technology Readiness Level 4, i.e., at the prototype development stage. **Conclusions**: Despite the limitation of a single database search, a surge in AI applications in ocular oncology after 2020 is evident. Most studies are in the model development stage, and few have been deployed in the real world for clinical implementation. Very few models have proven effective in real-world clinics and the community, holding promise for the future.

## Simple Summary

Most of the artificial intelligence (AI) work in ocular oncology has focused on the common eye cancers, such as uveal melanoma, retinoblastoma, and, to a lesser extent, orbital and eyelid tumors. As with other fields, the scope of AI in ocular oncology is also rapidly expanding, from a single PubMed-indexed work in 2001 to 35 in 2025. From analyzing fundus photographs to interpreting multimodal imaging to analyzing omics data, the scope of AI in ocular oncology is also rapidly evolving. In this review, we provide a comprehensive overview of the current literature on artificial intelligence in ocular oncology, highlighting the disease domains, problem statements addressed, technologies used, types of data input, predicted use cases, areas for improvement, and overall future directions.

## 1. Introduction

Ocular oncology, like many other fields of medicine, has undergone a major revolution since its formal recognition as a distinct field around the 20th century. It is a niche area of ophthalmology with unique challenges in every aspect of diagnosis, treatment, and surveillance. From anecdotal reports to global multicentric studies, from radical surgeries to protocol-based vision-preserving treatments, from histopathological diagnosis to rapid, non-invasive diagnosis, from morbid treatments such as enucleation and orbital exenteration to targeted, personalized therapy, from follow-up of families to pre-implantation genetic diagnosis, from fragmented care to multidisciplinary management, several advances have been made in the field of ocular oncology [1]. While we acknowledge the “human intelligence” of stalwarts in the field who have crystallized, defined, advanced, and transformed it [1], we must also recognize that “artificial intelligence” (AI) is revolutionizing ocular oncology in the 21st century [2]. Healthcare has been cited as the most promising application of AI [3], and this holds true for ocular oncology as well. For an ocular oncologist, it is essential to stay abreast of how AI is advancing the diagnosis, treatment, and, most importantly, the prognostication of patients with eye tumors. For clinician-scientists, knowledge of this evolving field also guides cutting-edge research to tackle complex problems in ocular oncology. Current review articles on AI in ocular oncology focus on specific tumor types, including uveal melanoma, retinoblastoma, intraocular tumors, ocular surface tumors, and ocular adnexal tumors [2,4,5,6,7,8,9,10,11,12], using varied methodology. In this major review, we provide a structured summary of the entire spectrum of AI research in ocular oncology and discuss future directions.

## 2. Materials and Methods

This scoping review included PubMed-indexed original articles in the English-language literature on the use of artificial intelligence in ocular oncology. The PRISMA flow diagram [13] was used to guide the identification, screening, and inclusion of articles from the PubMed database. A PubMed database search was performed on 9 February 2026, using MeSH terms “artificial intelligence”, “deep learning”, “machine learning”, “ocular oncology”, “ophthalmic oncology”, and “eye cancer.” The following search queries were provided:

(i) ((artificial intelligence[MeSH Terms]) OR (deep learning[MeSH Terms])) OR (machine learning[MeSH Terms]) AND ((eye cancer[MeSH Terms]) OR (ocular oncology[MeSH Terms])) OR (ophthalmic oncology[MeSH Terms]);
(ii) ((artificial intelligence[Title/Abstract]) OR (deep learning[Title/Abstract])) OR (machine learning[Title/Abstract]) AND ((ocular oncology[Title/Abstract]) OR (eye cancer[Title/Abstract])) OR (ophthalmic oncology[Title/Abstract]).

The full search strings were as follows:

(i) (“artificial intelligence”[MeSH Terms] OR “deep learning”[MeSH Terms] OR “machine learning”[MeSH Terms]) AND (“eye neoplasms”[MeSH Terms] OR ((“ocular”[All Fields] OR “oculars”[All Fields]) AND “neoplasms”[MeSH Terms]) OR ((“eye”[MeSH Terms] OR “eye”[All Fields] OR “ophthalmic”[All Fields] OR “ophthalmically”[All Fields] OR “ophthalmics”[All Fields]) AND “neoplasms”[MeSH Terms]));
(ii) (“artificial intelligence”[Title/Abstract] OR “deep learning”[Title/Abstract] OR “machine learning”[Title/Abstract]) AND (“ocular oncology”[Title/Abstract] OR “eye cancer”[Title/Abstract] OR “ophthalmic oncology”[Title/Abstract]).

The search output was input into Microsoft Excel^®^ for Mac (Version 16.107) to screen the records and remove duplicates. No additional grey literature was performed. All records were screened, and the abstracts of articles were retrieved and assessed for eligibility of inclusion, i.e., (i) original work, (ii) any form of artificial intelligence has been employed to solve a problem pertaining to ocular oncology, (iii) written in the English language, and (iv) published before January 2026. Entries of review articles, conference proceedings, commentaries on original work, work that did not employ AI, or not pertaining to ocular oncology were excluded. Articles not in English were excluded due to language translation logistics. All records were reviewed independently by VSV and SK, and any discrepancies were discussed (Table S1).

Data extracted for the review included the year of publication, the ocular pathology evaluated, and the purpose of AI use. In addition, a country label was assigned to each work based on the corresponding author’s geographic location to compare the volume of AI work in ocular oncology from different countries. Details reviewed from the AI model perspective included: learning strategy (supervised/semi-supervised/unsupervised), architecture type (classic machine learning (ML), convolutional neural network (CNN), or transformer), training level (training, internal validation, or external validation), algorithm type (explainable or Blackbox), patient data type (images, textual data, or a combination), dataset size, Clinical AI readiness evaluator (CARE) Technology Readiness Level [14], AI lifecycle phase [15,16], and AI performance metrics.

## 3. Results

**Figure 1:**
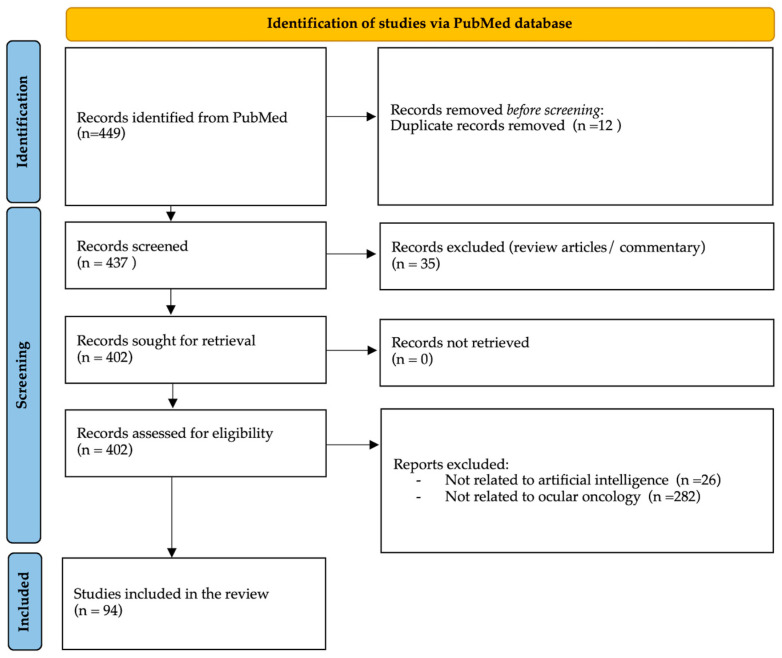
PRISMA flowchart.

A total of 450 records were identified using the search methodology described above. Ninety-four studies were included in the review through a screening process detailed in Figure 1 [17,18,19,20,21,22,23,24,25,26,27,28,29,30,31,32,33,34,35,36,37,38,39,40,41,42,43,44,45,46,47,48,49,50,51,52,53,54,55,56,57,58,59,60,61,62,63,64,65,66,67,68,69,70,71,72,73,74,75,76,77,78,79,80,81,82,83,84,85,86,87,88,89,90,91,92,93,94,95,96,97,98,99,100,101,102,103,104,105,106,107,108,109,110]. The complete list of included articles and overview parameters is provided in Table S1. PRISMA checklist see File S1.

### 3.1. Overview of AI Research in Ocular Oncology

**Figure 2:**
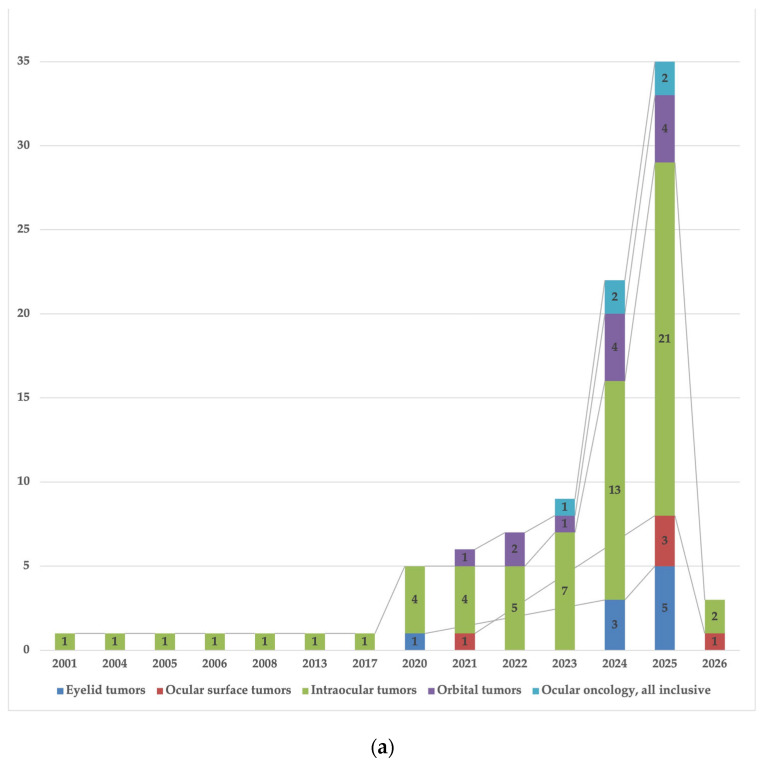

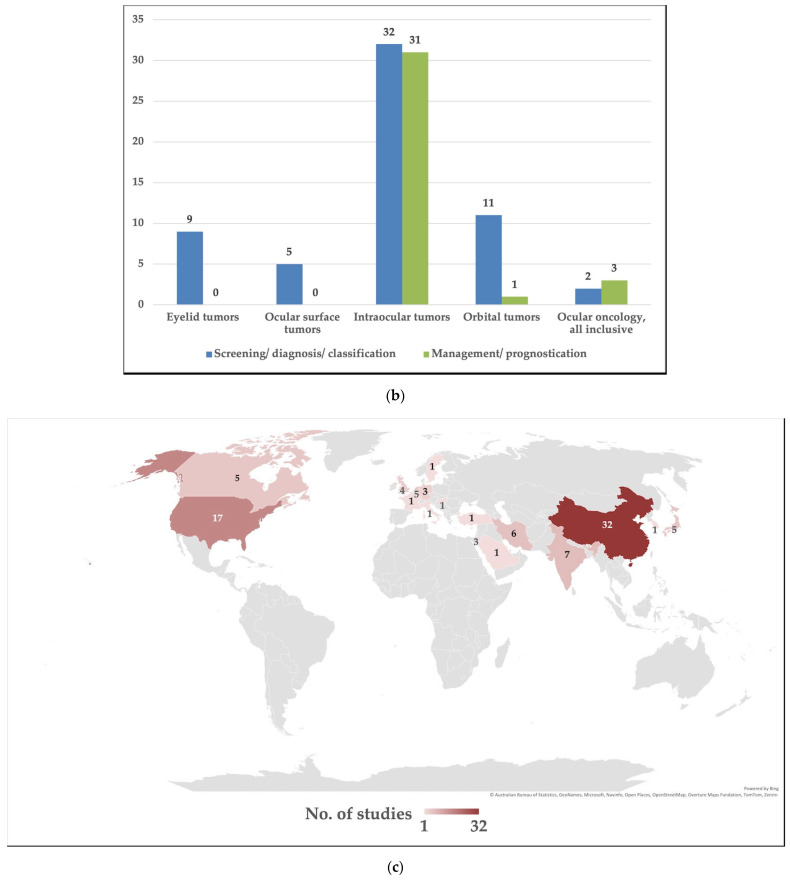
Temporal trend of artificial intelligence research in ocular oncology (**a**), distribution of focus domain and purpose of the studies (**b**), and geographic distribution of AI research in ocular oncology (**c**).

The trend in AI research in ocular oncology showed a temporal progression, with one research article in 2001 and 35 in 2025 [Figure 2a]. In total, 63 studies (70%) focused on intraocular tumors, 12 (13%) on orbital tumors, 9 (10%) on eyelid tumors, and 5 (5%) on ocular surface tumors. Five studies (5%) focused on all-inclusive, general ocular oncology. Within intraocular tumors, which comprised the major domain, 39 (41%) studies focused on uveal melanoma and its differentials (UM) and 14 (15%) focused on retinoblastoma (RB). A total of 59 (63%) studies employed AI models for screening, diagnosing, or classifying eye tumors and 35 (37%) examined management, including prognostication, in patients with eye tumors [Figure 2b].

#### 3.1.1. Geospatial Trends

Based on the geographic location of the research, the largest share of AI research in ocular oncology was from China (34%), followed by the USA (18%), India (7%), and Iran (6%) [17,18,19,20,21,22,23,24,25,26,27,28,29,30,31,32,33,34,35,36,37,38,39,40,41,42,43,44,45,46,47,48,49,50,51,52,53,54,55,56,57,58,59,60,61,62,63,64,65,66,67,68,69,70,71,72,73,74,75,76,77,78,79,80,81,82,83,84,85,86,87,88,89,90,91,92,93,94,95,96,97,98,99,100,101,102,103,104,105,106,107,108,109,110] [Figure 2c].

#### 3.1.2. Datasets

The AI models were exposed to a wide variety of datasets in these studies, with few employing a combination of data. Of the 71 studies that used images, 49 (70%) aimed to screen, diagnose, or classify tumors. Among 39 studies that used non-photographic data, 22 (56%) focused on management decisions or tumor prognostication.

**Figure 3:**
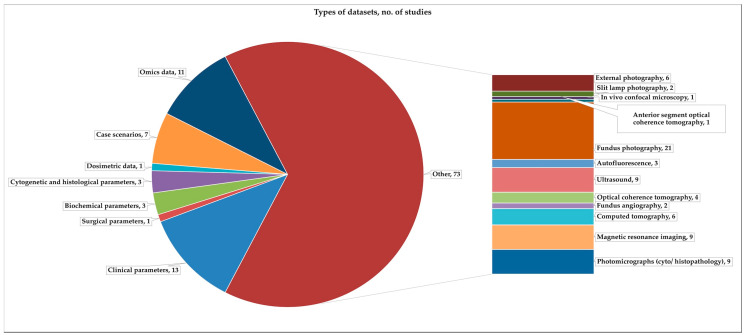
Types of datasets employed.

The datasets included images in most studies (*n* = 73, 78%). Fundus photographs were the most common image dataset and were used in 22% of the studies (*n* = 21). Other forms of images utilized included external photographs (*n* = 6, 6%), slit lamp photographs (*n* = 2, 2%), anterior segment optical coherence tomography scans (*n* = 1, 1%), in vivo confocal microscopy (*n* = 1, 1%), fundus autofluorescence photographs (*n* = 3, 3%), ultrasonographic images (*n* = 9, 10%), optical coherence tomography scans (*n* = 4, 4%), fundus angiogram images (*n* = 2, 2%), computed tomography (CT) scans (*n* = 6, 6%), magnetic resonance imaging (MRI) scans (*n* = 9, 10%), and photomicrographs of cytopathology or histopathology slides (*n* = 9, 10%). Datasets beyond imaging (non-photographic data) included clinical parameters (*n* = 13, 14%), surgical parameters (*n* = 1,1%), biochemical parameters (*n* = 3, 3%), cytogenetic and histological parameters (*n* = 3, 3%), radiation dosimetric data (*n* = 1, 1%), case scenarios as text input (*n* = 7, 7%), and omics data (*n* = 11, 12%) [Figure 3].

The dataset size was small (<500) in 51 studies, medium (500 to 2000) in 23 studies, and large (>2000) in 20 studies [Table S2]. With respect to the type of data input to the AI models, data at a single point in time were used in 88 studies and a combination of data from a single point (still ultrasound images) and over a time frame (ultrasound cine loops) was used in 1 study [73]. Six studies used data collected over a longitudinal period, with temporal analysis performed in three of them. The latter included studies that assessed: (i) regression patterns of choroidal melanoma from ultrasound images [88], (ii) an assessment of response to tebentafusp in metastatic uveal melanoma [89], and (iii) tumor growth estimation models [109].

A detailed summary of AI research in eyelid, ocular surface, orbital, and intraocular tumors is provided herewith.

### 3.2. AI Research in General Ocular Oncology

Five studies evaluated the performance of AI models in general ocular oncology [Table 1]. All these studies are relatively recent and have deployed commercially available AI models. Three of them provided text prompts to chatbots, and one provided a combination of image and textual data. One study deployed the Medios AI system (Remidio, Singapore, Singapore) in a real-world setting for patients with systemic oncology, but the model performed poorly in detecting tumor-related findings from fundus photographs [17,18,19,20,21].

### 3.3. AI Research in Eyelid Tumors

Nine studies examined the use of AI in the management of eyelid tumors [Table 2]. The specific use cases were: (i) identification of eyelid tumors and malignancies from external photographs, (ii) automation of histopathological diagnosis from whole slide images, (iii) diagnosis of malignancy through AI-based proteomic analysis, and (iv) enhancing trainees’ eyelid tumor diagnostic skills. Reasonable accuracy (>80%) was observed across all studies [22,23,24,25,26,27,28,29,30]. Hui S et al. have deployed the developed model as a smartphone-based application to aid self-diagnosis of eyelid tumors.

### 3.4. AI Research in Ocular Surface Tumors

The ocular surface is the most recent domain in which AI is being employed [Table 3]. Each work has explored AI’s utility across different datasets, i.e., anterior segment images, anterior segment optical coherence tomography scans, in vivo confocal microscopy images, and ultrasound biomicroscopy images. Most work has evaluated OSSN, and one study focused on identifying conjunctival melanoma. Accuracy for the diagnosis of ocular surface squamous neoplasia (OSSN) ranges from 75% to 90% and, for conjunctival melanoma, 97% [31,32,33,34,35].

### 3.5. AI Research in Orbital Tumors

Twelve studies [Table 4] employed AI to differentiate malignant from benign orbital lesions (*n* = 2), differentiate between two orbital pathologies (*n* = 7), and automate segmentation and measurement techniques for CT and MRI (*n* = 3). Datasets utilized include orbital imaging, histopathology images, and clinical data. The diagnostic accuracy ranges from 69% to 95% across different pathologies [36,37,38,39,40,41,42,43,44,45]. The dice coefficients for models aimed at automating/semi-automating segmentation and measurements in orbital imaging are at 70% to 80% [46,47].

### 3.6. AI Research in Intraocular Tumors

AI has been extensively researched for the screening, diagnosis, treatment and prognostication of intraocular tumors with a major focus on UM, followed by RB [48,49,50,51,52,53,54,55,56,57,58,59,60,61,62,63,64,65,66,67,68,69,70,71,72,73,74,75,76,77,78,79,80,81,82,83,84,85,86,87,88,89,90,91,92,93,94,95,96,97,98,99,100,101,102,103,104,105,106,107,108,109].

#### 3.6.1. Retinoblastoma

Fourteen studies employed AI for RB [Table 5]. Early studies focused on automating boundary detection on ultrasound and tumor segmentation on MRI [48,49]. Studies have utilized fundus photographs to train AI models to detect RB (*n* = 7), classify RB (*n* = 3), differentiate RB from mimickers (*n* = 1), and to monitor disease activity (*n* = 2) [50,51,52,53,54,55,56,57]. AI for prognosticating RB outcomes has recently been explored using MRI (*n* = 1), RNA profiling (*n* = 2), and metabolic profiling (*n* = 1) [58,59,60,61].

#### 3.6.2. Uveal Melanoma

UM is the most common primary intraocular tumor with significant morbidity and mortality. The majority of AI research in ocular oncology focuses on uveal melanoma, and the progress achieved is remarkable [62,63,64,65,66,67,68,69,70,71,72,73,74,75,76,77,78,79,80,81,82,83,84,85,86,87,88,89,90,91,92,93,94,95,96,97,98,99,100]. AI for uveal melanoma has advanced on two main fronts: (i) early detection with focus on screening, establishing an accurate diagnosis, differentiating from mimickers, and identifying high-risk features [Table 6] [62,63,64,65,66,67,68,69,70,71,72,73,74,75,76] and (ii) predicting outcomes [Table 7] [77,78,79,80,81,82,83,84,85,86,87,88,89,90,91,92,93,94,95,96,97,98,99,100].

Fundus photographs are the most used data type, with studies employing thousands of images to train and test models for detecting choroidal lesions. AI models’ accuracies range from 70% to 93% for detecting choroidal nevus or melanoma in fundus images [62,63,64,65,66,67,68,69,70,71,72]. Emmert N et al. developed a model that achieved 99% accuracy (in a validation cohort) in detecting choroidal lesions from ultrasonographic cine loops [73]. AI has also been used to detect BAP1 nuclear expression in histopathology slides of UM, to differentiate UM from other intraocular lesions on MRI, and to detect the presence of intraocular UM from tear sample liquid biopsy [74,75,76].

Prognosticating UM outcomes is a complex problem, and the ability of AI models to process and analyze diverse data types is an advantage over conventional analysis. Early studies utilized clinical data and multimodal imaging, and the recent literature shows an increased incorporation of bioinformatic data to assign risk signatures to UM [Table 7]. Overall, the maximum accuracy of AI models for predicting the malignant transformation of choroidal nevi is 91%, for predicting metastasis in UM is 87%, and for predicting death from UM is 93%, based on clinical data, multimodal imaging, and cytogenetic and histopathological data [77,78,79,80,81,82,83,84]. Expanding use cases for AI in UM include enabling treatment decisions, predicting regression patterns and local recurrence after plaque radiotherapy, predicting response to targeted therapy in metastatic UM, and identifying high-risk signatures by incorporating bioinformatics data [85,86,87,88,89,90,91,92,93,94,95,96,97,98,99,100].

#### 3.6.3. Other Intraocular Tumors

Other specific intraocular tumors where AI has been employed include ocular metastasis (*n* = 2) and primary vitreoretinal lymphoma (*n* = 2) [101,102,103,104]. Other studies focused on diagnosing various intraocular diseases, including tumors, assessing the response of AI chatbots to ocular oncology case scenarios, developing comprehensive intraocular datasets for AI model development, developing a model to guide proton therapy, and developing tumor segmentation techniques [105,106,107,108,109,110]. These are summarized in Table 8.

### 3.7. Evaluation of AI Models and Critical Appraisal

Most studies worked on developing AI models (*n* = 83, 88%), of which two reached a deployment stage [Table S2]. These studies were reviewed for learning strategy, architecture type, algorithm type, training level, risk of overfitting, and CARE technology readiness levels. A supervised learning strategy was employed in 75 studies (80%); a semi-supervised learning strategy in 4 (4%); a self-supervised and supervised learning strategy in 2 (2%); a combination of unsupervised and semi-supervised learning strategy in 1 (1%); and a combination of unsupervised and supervised learning strategy in 1 (1%). Machine learning architecture was employed in 36 studies (38%), deep learning in 39 (41%; CNN, *n* = 32 (34%); transformer, *n* = 2 (2%); combination, *n* = 5 (5%)), and a combination of machine learning and deep learning in 8 studies (9%). Algorithms were black-box in 33 studies (35%) and explainable in 50 studies (53%). Internal validation in 66 studies (70%) and external validation of the developed models was performed in 13 studies (14%). Four studies describing the automation of image-processing techniques were not validated in clinical cohorts.

After excluding the 4 studies that solely described techniques, the risk of AI model overfitting was assessed in 79 studies. Notably, the risk of overfitting could not be assessed in 44 studies because the authors did not report training metrics alongside test metrics of the AI models. It was evaluated as likely to be low in 5 studies, likely high in 1 study based on the methodology, and low in 30 studies that objectively report training, test, and validation metrics.

Few studies evaluated or incorporated pre-existing models (*n* = 11, 12%). Seven (7%) of these utilized chatbots/large language models. Among the remaining four studies that utilized pre-existing AI models and platforms, these were applied to diagnosing ocular retinal pathology in cancer patients (*n* = 1; Medios AI (Remidio, Singapore, Singapore)) [21], monitoring response to treatment on whole-body positron emission tomography scans (*n* = 1, RECOMIA platform) [89], generating eyelid tumor images for educational purposes (*n* = 1; LoRA) [30], and to develop a statistical prognostic model for uveal melanoma using transcriptomic data (*n* = 1; CIBERSORT) [100].

Evaluating the stage of the AI life cycle in the literature, most studies (*n* = 81, 86%) are in the model development stage, and few (*n* = 13, 14%) are in the model evaluation phase. Of note, only 2 studies that initiated model development deployed their models, while the other 11 studies used pre-existing models, with chatbot/large language models comprising the majority.

From a clinical standpoint, 83 studies were eligible for CARE assessment. Eleven studies that were not included in this assessment were: (i) Prompts to Chatbots (*n* = 6), (ii) CIBERSORT employed to develop a statistical prognostic model (*n* = 1), and (iii) AI-based automation of image-processing techniques (*n* = 4). Studies were categorized as technology readiness level 3 (proof-of-concept, *n* = 9), level 4 (prototype development, *n* = 59), level 5 (machine learning capability for clinical use, *n* = 2), level 7 (integrations, *n* = 11), level 8 (mission ready, *n* = 1), and level 9 (deployed and live, *n* = 1).

## 4. Discussion

The performance of an AI model depends on the availability of large, high-quality datasets [111]. The diagnosis and management of ocular tumors consider several parameters, including, but not limited to, demographics, clinical features, multimodal imaging, and tissue biopsies [112]. Each of these contributes to a large volume of data, thereby creating immense potential to utilize AI to address diagnostic and therapeutic challenges in ocular oncology. This potential is being increasingly tapped over the years, as reflected in the timeline of AI research in ocular oncology [Figure 2].

### 4.1. AI Research in Different Domains in Ocular Oncology

The incidence of UM ranges from 316 per year in the Black population to 4351 per year in the White population and is a significant burden in terms of mortality and morbidity [113]. Accordingly, most AI research in oncology is in UM. A review of AI research at UM offers insights into progress and indicates it is headed in the right direction [62,63,64,65,66,67,68,69,70,71,72,73,74,75,76,77,78,79,80,81,82,83,84,85,86,87,88,89,90,91,92,93,94,95,96,97,98,99,100]. Early studies employed AI to predict metastatic and survival outcomes in UM [79,80,81]. Over time, the scope has expanded to include identifying choroidal nevi at risk for malignant transformation, distinguishing UM from other choroidal lesions, and the various simulating lesions [63,64,65,66,67,68,69,70,71,72,73,77,78]. In addition to enabling early and accurate diagnosis of UM, AI models have greatly transformed the landscape of UM prognostication [79,80,81,82,83,84,85,86,87,88,89,90,91,92,93,94,95,96,97,98,99,100]. With excellent local tumor control outcomes [113], the recent focus appears to be on multimodal imaging and the integration of bioinformatic analysis to predict metastasis and survival in UM [92,93,94,95,96,97,98,99,100]. It is worth noting that no studies have yet explored AI’s ability to prognosticate globe- and vision-salvage outcomes; exploring this in future studies would be useful.

Aside from UM, a significant fraction of AI research focuses on the RB domain, as it is the most common primary intraocular tumor in children [114]. Chai MI et al. laid the foundation for the use of AI in RB by developing a neural network model for automatic boundary detection of retinoblastoma in 3D ultrasound images [48]. Following this, a major advance in the work has been in the detection and classification of RB, its differentiation from mimickers, and the monitoring of tumor activity from fundus photographs. At present, AI models can achieve acceptable accuracy across all three case scenarios [49,50,51,52,53,54,55,56,57]. Further, as with UM, the focus in RB is also evolving towards predicting outcomes to potentially guide globe-salvage decisions [58,59,60,61]. However, the evidence on the use of AI to predict outcomes in RB is nascent.

Evidence shows that AI may improve management of patients with other intraocular tumors, such as ocular metastases and intraocular lymphoma [105]. AI research on eyelid, ocular surface, and orbital tumors is more recent and continues to evolve [22,23,24,25,26,27,28,29,30,31,32,33,34,35,36,37,38,39,40,41,42,43,44,45,46,47].

### 4.2. Geospatial Trends

The largest contribution of AI research in ocular oncology is from China (34%), followed by the USA (18%), India (7%), and Iran (6%) [17,18,19,20,21,22,23,24,25,26,27,28,29,30,31,32,33,34,35,36,37,38,39,40,41,42,43,44,45,46,47,48,49,50,51,52,53,54,55,56,57,58,59,60,61,62,63,64,65,66,67,68,69,70,71,72,73,74,75,76,77,78,79,80,81,82,83,84,85,86,87,88,89,90,91,92,93,94,95,96,97,98,99,100,101,102,103,104,105,106,107,108,109,110]. Most of the AI work on eyelid and orbital tumors originates in China; retinoblastoma in China, followed by India; and uveal melanoma in the USA and China [17,18,19,20,21,22,23,24,25,26,27,28,29,30,31,32,33,34,35,36,37,38,39,40,41,42,43,44,45,46,47,48,49,50,51,52,53,54,55,56,57,58,59,60,61,62,63,64,65,66,67,68,69,70,71,72,73,74,75,76,77,78,79,80,81,82,83,84,85,86,87,88,89,90,91,92,93,94,95,96,97,98,99,100,101,102,103,104,105,106,107,108,109,110], which the authors speculate that it indirectly indicates tumor burden and resource allocation across regions. Analysis of eye cancer data from the Global Burden of Disease Study 2021 reveals considerable heterogeneity in the burden of eye cancer across 204 countries. A higher mortality is noted from eye cancer in low sociodemographic index countries, likely due to delayed diagnosis and inadequate treatment, especially in Sub-Saharan Africa [115]. At the time of drafting this manuscript, there is no work on AI in ocular oncology in this region, either in developmental or deployable phases. This highlights a significant gap where the deployment of scalable AI technology can improve outcomes.

It is also worth noting that research and AI models/tools developed in advanced countries may not be transferable to other countries due to (i) differences in disease burden (e.g., uveal melanoma is extremely rare in Africans [113], and there is likely no benefit of deploying a model addressing any aspect of uveal melanoma care in the region) and (ii) differences in the features of data that is presented to the model at the time of training (e.g., fundus color is known to influence an AI model’s ability to detect retinoblastoma tumors across different races [51]).

On the other hand, validation of models in a different geospatial cohort has its own advantages. Exposing the model to data from different geographical populations ensures robustness and real-world utility. Li et al. developed and validated TrajVis, an AI-assisted clinical decision support system tool for precision management of chronic kidney disease. The model was built on electronic medical records from the North Carolina population (Atrium Health Wake Forest Baptist Translational Data Warehouse) and the Indiana population (Indiana Network for Patient Care 30 research database) to reveal chronic kidney disease progression trajectories. Geospatial network databases such as these serve as valuable resources that improve the model’s performance in precision management [116]. In a study that focuses on gene expression prediction from whole slide images, training on multiple cancer datasets demonstrated superior performance [117]. Thus, both applicability to indigenous populations and versatility are important for an AI model’s real-world use. In addition, developing a diverse, all-inclusive, de-identified database for ocular cancers can benefit future AI work in the field.

### 4.3. Data and Technology

Most studies employed small datasets (*n* = 51, 54%), and only 20 (21%) studies utilized large datasets [17,18,19,20,21,22,23,24,25,26,27,28,29,30,31,32,33,34,35,36,37,38,39,40,41,42,43,44,45,46,47,48,49,50,51,52,53,54,55,56,57,58,59,60,61,62,63,64,65,66,67,68,69,70,71,72,73,74,75,76,77,78,79,80,81,82,83,84,85,86,87,88,89,90,91,92,93,94,95,96,97,98,99,100,101,102,103,104,105,106,107,108,109,110]. While most research focuses on databases of static images, few studies have explored the dynamism of these images from diagnostic and prognostic perspectives. Emmert et al. describe automated detection and assessment of choroidal mass lesions using still ultrasound images and ultrasound cine loops. They noted 84% accuracy on still images and 99% on cine loops in detecting choroidal mass lesions. Another role of dynamic or serial imaging lies in longitudinal follow-up and prediction. Few studies assessed this through: (i) regression patterns of choroidal melanoma from serial ultrasound images [88], (ii) an assessment of response to tebentafusp in metastatic uveal melanoma through serial whole-body positron emission tomography imaging [89], and (iii) intraocular tumor growth estimation models based on optical coherence tomography imaging [109]. Thus, dynamic assessment of images appears underexplored, and this aspect can certainly be improved in future studies.

A variety of AI models have been employed for the detection of ocular tumors, including supervised, unsupervised, and semi-supervised learning models, as well as combinations of these. Both machine learning and deep learning architectures are equally popular, with a combination of both used in a few studies. It is interesting to note that explainable AI algorithms were used in >50% of the studies. A significant barrier to adopting AI into clinical practice is the inability to “trust” the AI’s output or decision. This is typically the case with traditional black-box architectures such as artificial neural networks. In contrast, explainable AI provides the user with transparency into the output. While both architectures have their respective pros and cons, in the clinical setting, explainable AI is increasingly preferred due to the possibilities of: (i) verifying the presence of noise in the data, (ii) understanding the reason for decision errors made by AI, (iii) gaining better insights into the model’s learnings from the data, and (iv) providing better communication to patients when AI is employed for aiding clinical decisions or sought for second opinions [118]. In the current era, commercially available chatbots such as ChatGPT are black-box architectures [119]. Although few studies have evaluated the utility of chatbots in ocular oncology, their role may be limited due to their opacity.

### 4.4. Stage of AI Models, Clinical Readiness, and Challenges to Deployment in the Real World

To assess the impact of developing an AI model, it is essential to review its lifecycle phases [15,16]. The lifecycle of an AI model comprises data creation, data acquisition, model development, model evaluation, and model deployment phases [15]. DeSilva et al. simplified this into three phases: design, develop, and deploy [16]. The importance of highlighting these phases is that, until a model is deployed, its utility in real-world scenarios remains uncertain.

Upon reviewing the literature on AI in ocular oncology, only 13/94 research articles discuss the deployment of AI models. Of these, 11 were pre-existing models and 2 progressed from development to deployment. Seven studies utilized chatbots/large language models. Although evaluating AI-based chatbots provides insights into their accuracy, the utility of this information for clinical diagnosis or management is limited [17,18,19,20,65,106]. Among the remaining four studies that utilized pre-existing AI models and platforms, the following provide few insights: (i) work by Das et al. shows that the MediosAI system performs poorly in real-world fundus evaluation from an ocular oncology standpoint [21] and (ii) Tabuchi et al. demonstrate the utility of an image-generating AI model (LoRA) in improving learning outcomes [30].

The two studies that developed AI models for clinical problems and advanced them to a deployment stage are discussed here:

Hui S et al. demonstrate the development of an AI model for diagnosing eyelid tumors and its deployment via a smartphone-based mobile application, enabling “self-diagnosis” of eyelid lesions. However, the data on its utility in the community has yet to be determined [25].

Li S et al. developed a random forest model to diagnose primary vitreoretinal lymphoma from complete blood count parameters. Following internal and external validation, the model was deployed in hospital- and community-based cohorts to assess its real-world performance. In the community-based cohort, a total of 511,786 patients were screened. Among the 22 individuals identified by the model as high risk for primary vitreoretinal lymphoma and referred to the Ophthalmology service, 13 were confirmed to have PVRL. This work provides an excellent case study for employing AI with an inexpensive lab test to enable disease detection in the community [104].

From a clinical standpoint, most studies were categorized as CARE technology readiness levels 3 and 4, i.e., proof of concept and prototype development. Thus, most AI work in ocular oncology is in the model development phase, a major limitation. Deployment of models in real-world scenarios also enables assessment of cost-effectiveness, which would inform policies and protocols.

Another factor that determines the real-world application of AI is its performance during external validation. Only 14% of studies report external validation of AI models involving multiple ocular oncology centers. This leads to limited dataset diversity, which can result in possible underperformance of the model in the real world. Further, nearly 50% of the studies do not provide performance metrics for the training, validation, and test cohorts, thereby preventing assessment of the risk of overfitting.

Other challenges to the implementation of AI in ocular oncology practice that are likely to surface with research on a larger scale and during implementation phases include those of an ethical nature and safety.

### 4.5. Limitations

Limitations of this work include (i) the use of a single database search, (ii) work published in the English language only, (iii) an all-inclusive criterion of AI work in ocular oncology, hence the heterogeneity across studies, (iv) and the risk of bias from the studies could be assessed only to a certain extent, limited by the data availability.

### 4.6. Future Direction

While cutting-edge AI research to improve diagnosis in eyelid and orbital tumors and improve patient survival and eye salvage in uveal melanoma and retinoblastoma continues, efforts should focus on deploying existing models for screening and early detection, especially in countries with low sociodemographic indices; however, existing models should be validated before deployment to the public domain. International collaboration with ethical data sharing practices should advance this effort.

## 5. Conclusions

AI in ocular oncology is showing significant progress, particularly in UM and RB. However, most AI models are at a technology readiness level of proof of concept or prototype development, and the focus should be on achieving clinical implementation. In addition, the geospatial disparities need to be addressed to improve the reach of these evolving technologies. From the authors’ experience in developing an AI model for the detection of RB [50,51,52], the way forward appears to be: (i) large-scale external validation through prospective multinational data sharing. This should improve the versatility of the model across different populations. (ii) Collaborations with technology and industry partners to develop a deployable tool and (iii) involvement of international eyecare organizations for taking an AI-driven technological tool to the global communities.

## Figures and Tables

**Table 1:** Summary of AI research in general ocular oncology.

Citation	Objective	Dataset	AI Methods	Performance Metrics
Sakai D et al., 2023 [17]	Assess the accuracy of ChatGPT responses (answers) to prompts (questions from past board examinations in ophthalmology)	21 questions in orbit and oncology	ChatGPT-3.5 and ChatGPT-4	Correct answer rate: ChatGPT-3.5: 24% ChatGPT-4: 71%
Tailor PD et al., 2024 [18]	Determine the appropriateness of chat-based AI model for ophthalmology scenarios including ocular oncology	20 ocular oncology questions (of 192 ophthalmology questions)	ChatGPT	Median % of appropriate answers (graded as appropriate, inappropriate or unreliable by 1 subspecialists): 90% for ocular oncology
Mihalache A et al., 2024 [19]	Evaluate ChatGPT’s ability to process case scenarios with multimodal imaging	18 image-based questions and 11 non-image-based questions in ocular oncology Input: Text, fundus photographs, OCT, autofluorescence, fundus angiography, visual fields, A-scan	ChatGPT	Ocular oncology: Better accuracy for non-image-based questions (91%) than image-based questions (61%)
Das D et al., 2025 [20]	Assess the response of AI chatbots to ocular oncology related queries	5 case scenarios with textual descriptions of ocular findings, systemic findings and imaging findings	AI chatbots: ChatGPT-4o, DeepSeek v3, and Gemini 2.0	Average correctness score (all 3 models): 3.4/5 Average reliability score: ChatGPT-4o: 3.2 DeepSeek v3: 2.8 Gemini 2.0: 3.0
Das D et al., 2025 [21]	Study the accuracy of Medios AI in a real-world scenario for patients with systemic malignancies	Fundus photographs (98 patients’ malignancies, eyes, 196 images)	Medios AI	0% of 8 patients with leukemic retinopathy were correctly diagnosed

GPT = Generative Pre-trained Transformer; AI = artificial intelligence; OCT = optical coherence tomography.

**Table 2:** Summary of AI research in eyelid tumors.

Citation	Problem Addressed	Dataset	AI Methods	Performance Metrics
Zhang J et al., 2025 [22]	Differentiating eyelid basal cell from sebaceous carcinoma using external photographs	External photographs (171 = eyelid sebaceous carcinoma and 199 = eyelid basal cell carcinoma)	ResNet50- based deep learning model	Accuracy: Overall: 89% Basal cell carcinoma: 92% Sebaceous carcinoma: 86%
Fan W et al., 2025 [23]	Differentiating benign and malignant eyelid tumors	External photographs (397 = malignant tumor, 449 = benign tumor, 405 = control (no tumor))	Model developed based on various convoluted neural networks	VGG16: 75% ResNet50: 79% Efficient-Net: 81% Inception-V4: 79% Dual VGG16: 79% Dual ResNet50: 81% Dual Efficient-Net: 81% Dual Inception-V4: 84%
Zloto O et al., 2025 [24]	Distinguishing between benign and malignant eyelid lesions	External photographs of eyelid lesions (373 = benign, 186 = malignant)	EfficientNet-B5 convolutional neural network	Sensitivity: 94% Specificity: 92%
Hui S et al., 2025 [25]	Enabling self-diagnosis eyelid tumors	External photographs (*n* = 1195)	“Intelligent Eyelid Tumor Screening” developed from YOLOv5 and Efficient-Net v2-B architectures	Accuracy 92%
Li Z et al., 2022 [26]	Distinguishing between benign and malignant tumors	External photographs (*n* = 1417 images with 1533 tumors; 1161 = benign; 372 = malignant)	DenseNet121 algorithm	Accuracy 82%
Jiang Z et al., 2024 [27]	Automating the diagnosis of eyelid malignant melanoma from histopathology slides	Histopathological images (*n* = 312 whole slide and 32,680 patch level images from public dataset)	Self-supervised learning-based framework with integration of squeeze-excitation attention structure and a feature-projection structure	Accuracy: 86% at patch level and 92% for whole slide images
Wang L et al., 2020 [28]	Automating the diagnosis of eyelid malignant melanoma from histopathology slides by a deep learning system	Histopathological images (*n* = 155 whole slide and 225,230 patch level images from public dataset)	VGG16 network	Accuracy: 95% at patch level and 98% for whole slide images
Wang L et al., 2024 [29]	Classification of eyelid tumors	Proteomic data from formalin fixed paraffin embedded tissues (*n* = 233, discovery cohort), 99 = independent cohort)	Deep learning system developed using computer vision and neural networks, trained using 13 proteins	Accuracy: Normal vs. tumor: 95% Non-malignant vs. malignant: 90%
Tabuchi H et al., 2024 [30]	Improving eyelid tumor diagnostic skills of optometrists	55 optometry and vision science students, randomized to AI-based training (118 = sebaceous carcinoma images, 1046 = chalazion images) and conventional training	Image generation AI model	AI-based training comparable with conventional video lecture training

AI = artificial intelligence.

**Table 3:** Summary of AI research in ocular surface tumors.

Citation	Problem/Challenge	Dataset	AI Methods	Performance Metrics
Yoo TK et al., 2021 [31]	Detection of conjunctival melanoma	Anterior segment images from the web, for the diagnoses, pterygium, conjunctival nevus, melanosis, and melanoma	Generative adversarial networks (GAN) and GoogleNet, InceptionV3, NASNet, ResNet50, and MobileNetV2 architectures	Accuracy: MobileNetV2 with GAN-based augmentation: 97%
Kozma K et al., 2025 [32]	Differentiate OSSN from other pathology	In vivo confocal microscopy images (*n* = 2274)	ResNet50V2, Yolov8x, and VGG19.	Accuracy: 90%
Ramezani F et al., 2025 [33]	Differentiate OSSN from pterygium	Slit lamp photographs (77 = OSSN, 85 = pterygium)	Python-based transfer learning approach, EfficientNet B7 GoogleNet	Accuracy: 94%
Greenfield JA et al., 2025 [34]	Differentiate OSSN from pterygium and pingecula	Anterior segment optical coherence tomography scans (*n* = 108,780 scans of 4601 patients)	ViT- supervised model	Accuracy: 97%
Serbest Ceylanoglu K et al., 2026 [35]	Differentiate OSSN from other pathologies	Ultrasound biomicroscopy images (201 = benign lesions; 381 = OSSN)	Xception, convolutional neural network	Accuracy 75%

AI = artificial intelligence, OSSN = ocular surface squamous neoplasia.

**Table 4:** Summary of AI research in orbital tumors.

Citation	Problem/Challenge	Dataset	AI Methods	Performance Metrics
Huang W et al., 2025 [36]	Distinguishing orbital lesions through radiomics	CT images (*n* = 139 patients)	Logistic regression, Naive Bayes Classifier, support vector machine, Extra Trees Classifier, multilayer perceptron	Accuracy 83%
O’Shaughnessy E et al., 2024 [37]	Distinguishing between benign and malignant orbital tumors	MRI (*n* = 113 patients)	Python, Hyperopt, SHAP	Accuracy 81% (10-feature signature)
Li J et al., 2025 [38]	Distinguishing between IgG4 related orbital disease and orbital MALT lymphoma	Histopathology images (*n* = 214 patients)	Pyramidomics, LASSO regression model, support vector machine, KNN, RF, XGBoost, MLP, Naive Bayes	Accuracy 82%
Tagami M et al., 2024 [39]	Distinguishing IgG4-related orbital disease from orbital MALT lymphoma	Histopathology images of target regions (*n* = 1270 patches from 127 patients)	Python, PyTorch, EVA, ViT, EfficientNet, Densenet 121, Resnet50, Vgg16	Accuracy 73%
Zhang H et al., 2025 [40]	Distinguishing between idiopathic orbital inflammation and orbital lymphoma	MRI (*n* = 97 patients)	Attention-based fusion model, handcrafted features, convolutional auto-encoder, and bag-of-features	Accuracy 82%
Xie X et al., 2022 [41]	Distinguishing idiopathic orbital inflammation from orbital lymphoma	Clinical parameters, MRI images (*n* = 105 patients)	Convolutional neural networks and multimodal fusion layers	Accuracy 95%
Hou Y et al., 2021 [42]	Distinguishing idiopathic orbital inflammation from orbital lymphoma	Contrast-enhanced MRI of 56 patients (28 = idiopathic orbital inflammation, 26 = MALT lymphoma)	Bag-of-features based radiomics, Support vector machine	Accuracy 81%
Tagami M et al., 2023 [43]	Distinguishing conjunctival lymphoma from orbital lymphoma	Histopathology images (990 patch images from 99 patients)	Logistic regression, k-nearest neighbor method, support vector machine with linear kernel, support vector machine with radial basis function kernel, decision tree, random forest, gradient tree boosting models	Accuracy 85%
Tooley AA et al., 2022 [44]	Distinguishing intradiploic dermoid from epidermoid cysts	Patient parameters from published data (55 = intradiploic cysts)	Weka used for model creation. Algorithms: Kstar, Neural network, Logistic Regression, Gradient Descent, Naïve Bayes, Decision Tree	Accuracy: Kstar 76% Neural network 69%
Mitchell MB et al., 2025 [45]	Semi-automating segmentation techniques	CT, MRI	3D slicer	Not validated
Wang G et al., 2024 [46]	Segmenting and measuring orbital lymphoma tumor volume	MRI (*n* = 180 patients)	nnU-net model	Dice similarity coefficient 80%
Wang K et al., 2024 [47]	Semi-supervised segmentation method for orbital tumors on computed tomography images	CT images (*n* = 55 patients, 602 images)	Multi-scale consistent self- training network	Dice similarity coefficient 70%

CT: computed tomography; LASSO: least absolute shrinkage and selection operator; MRI: magnetic resonance imaging; SHAP: SHapley Additive exPlanation.

**Table 5:** Summary of AI research in retinoblastoma.

Citation	Problem/Challenge	Dataset	AI Methods	Performance Metrics
Chai MI et al., 2001 [48]	Automatic boundary detection of retinoblastoma	3D ultrasound, 463 data points and 127 validation points	Neural networks	Not validated
Strijbis VIJ et al., 2021 [49]	Automate RB tumor segmentation on MRI	MRI (*n* = 40 patients)	Multi-view convolutional neural networks	Dice similarity coefficient for tumor 91%
Kaliki S et al., 2023 [50]	Detection and classification of RB	Fundus photographs (*n* = 771 images of 109 eyes)	OpenCV, Mobile-Net v2 SSD-based deep learning model	Accuracy (detection) 74.2%
Vempuluru VS et al., 2025 [51]	Detection and classification of RB	Fundus photographs (*n* = 1266 images of from 173 eyes)	OpenCV, Mobile-Net v2 SSD-based deep learning model	Accuracy (detection) 85%
Vempuluru VS et al., 2024 [52]	Detection and classification of RB in a multiracial cohort	Fundus photographs (*n* = 2473 images)	OpenCV, Mobile-Net v2 SSD-based deep learning model, XGBoost ML model	Accuracy (detection) 94%
Aldughayfiq B et al., 2023 [53]	Development of an explainable AI model for detection of RB	Fundus photographs (400 = RB, 400 = no RB)	Inception V3 architecture model, LIME, SHAP techniques	Accuracy 97%
Hu Y et al., 2025 [54]	Development of a model for detect and assess tumor activity	Fundus photographs (3730)	RB-Care from YOLOv8	Accuracy 97% Segmentation: 96% to 100% Classification: 98%
Cruz-Abrams O et al., 2025 [55]	Development of a model to distinguish RB from pseudo RB	Fundus photographs (2882 image = RB, 1980 = pseudo RB, 840 = normal fundus)	ResNet-18, ResNet-34, ResNet-50, ResNet-101, ResNet-152, and a Vision Image Transformer	Accuracy 97%
Rahdar A et al., 2023 [56]	Automation of segmentation of RB tumors in fundus photographs	Fundus photographs (4200 images)	Gaussian Mixture Model	Accuracy 93%
Zhang R et al., 2023 [57]	Development of a model to detect the presence and activity of RB	Fundus photographs (47,503 images for development and 2032 for prospective validation)	Deep Learning Assistant for Retinoblastoma Monitoring algorithm developed from ResNet-50 and InceptionV3	Accuracy Normal vs. active RB: 99% Stable vs. active RB: 93%
de Bloeme CM et al., 2024 [58]	Differentiate MYCN amplified RB based on MRI radiomics	MRI of 98 patients with unilateral RB	Univariate feature selection, data oversampling (duplicating MYCN cases), logistic regression classifier, SHAP analysis	Area under curve 0.78 to 0.81
Ye R et al., 2024 [59]	Construction of a risk model based on prognostic genes in RB and osteosarcoma	RNA sequences and clinical data of patients with RB and osteosarcoma from databases	Univariate Cox regression analysis, LASSO, SVM, Random Forest	SVM method worked the best
Liu W et al., 2022 [60]	Develop an RB monitoring platform by aqueous humor metabolic finger printing	Aqueous humor samples (*n* = 66)	Logistic regression, LASSO, tree, random forest	Accuracy 85% (7-biomarker panel)
Li Z et al., 2013 [61]	Identify prognostic biomarkers through predicting cancer-related genes based on GO terms and KEGG pathways.	Transcriptomic sequences of 119 RB genes and 5500 non-RB genes	Dagging, maximum relevance minimum Redundancy, incremental feature selection	Accuracy, 88%

AI: artificial intelligence, LASSO: least absolute shrinkage and selection operator; LIME: Local Interpretable Model-Agnostic Explanations; MRI: magnetic resonance imaging, RB: retinoblastoma; SHAP: SHapley Additive explanation; SVM: Support Vector Machine.

**Table 6:** Summary of AI research in screening and diagnosis of uveal melanoma and related lesions.

Citation	Problem/Challenge	Dataset	AI Methods	Performance Metrics
Suri H et al., 2025 [62]	Classification of risk factors for the development of choroidal melanoma	Fundus photographs (404 eyes)	RETFound (Vision Transformer), Multilayer Perceptron	Area under curve Diameter > 5 mm: 93% Thickness > 2 mm: 84% Orange Pigment: 70% Subretinal Fluid: 77% Low Internal Reflectivity: 82%
Tasso V et al., 2025 [63]	Distinguishing UM from other pigmented fundus lesions	Fundus photographs (*n* = 864; uveal melanoma, choroidal nevi, CHRPE)	ResNet-50 backbone; CV models: classification, detection, segmentation	Area under curve (segmentation): 95%
Wu Y et al., 2025 [64]	To develop a model to distinguish uveal melanoma from choroidal hemangioma and metastatic carcinoma	Fluorescence angiography, indocyanine green angiography, ocular ultrasound images (750 patients: 542 with melanoma, 128 with hemangioma, and 80 with metastatic carcinoma)	Multimodal Medical Concept Bottleneck Model	Accuracy 90%
Sabazade S et al., 2025 [65]	Differentiating small choroidal melanoma from nevi	Fundus photographs (*n* = 25),	MOLES system (≥3) vs. MEL*AI*noma (U-net + Random Forest)	Accuracy MOLES system 57%. MEL*AI*noma 88%
Laycock E et al., 2025 [66]	Detecting choroidal melanocytic lesions	Fundus photographs (*n* = 388 images from 194 patients)	Deep Learning/Logistic Regression	Accuracy 77%
Eshragh M et al., 2024 [67]	Improving choroidal nevus position identification	Fundus photographs (*n* = 253)	U-net, Residual U-net, Attention U-net, voting-based Ensemble	Dice Coefficients: 88% (Ensemble)
Crump RT et al., 2025 [68]	Classification and segmentation fundus images with choroidal nevi	Fundus photographs (*n* = 591)	VGG-16, YOLOv8, ResNet 50, U-net	Accuracy (YOLOv8): 93%
Dadzie A et al., 2022 [69]	Differentiating choroidal melanoma from nevi	Fundus photographs (438 patients, 798 images)	DenseNet121 CNN, transfer learning with intermediate color channel fusion	Accuracy 89%
Ma J et al., 2024 [70]	Automating tumor segmentation on fundus photography with machine learning	Fundus photographs (*n* = 525)	DeepLabv3	Dice coefficients: uveal melanoma 86%, choroidal nevi 81% congenital hypertrophy of the retinal pigmented epithelium 85%
Jackson M et al., 2024 [71]	Differentiating between choroidal melanoma and nevus	Fundus photographs, fundus auto fluorescence (*n* = 4255 patients; 28,373 images)	RETFound (retrained)	Accuracy Healthy 83% Nevus 76% Uveal melanoma 86%
Mehta NN et al., 2025 [72]	Differentiating between choroidal melanoma and nevus	Demographic data, fundus photographs, autofluorescene, OCT, ultrasound B scan	ChatGPT4.0, GeminiAdvanced1.5Pro, PerplexityPro	Accuracy ChatGPT4.0: 31% GeminiAdvanced1.5Pro: 73% PerplexityPro: 65%
Emmert N et al., 2025 [73]	Automating detection and measurement of choroidal lesions on ultrasonography B scan	Ultrasound B scan (*n* = 1822 still images, 130 cine loops (+) *n* = 554 still images for validation)	EfficientNet-B3, U-Net++	Accuracy Stil images: 84% Cine loops: 99%
Zhang H et al., 2020 [74]	Detection of nuclear BAP1 expression on whole slide images of uveal melanoma	Histopathology slides (*n* = 140)	ResNet-18 (deep CNN, U-Net (auto-encoder-decoder)	Accuracy (slide level): 84%
Su Y et al., 2020 [75]	Differentiating choroidal melanoma from other intraocular disease	MRI (245 patients)	Logistic regression, multilayer perceptron, support vector machine	Accuracy 76 to 86%
Rakhshandeh H et al., 2025 [76]	Detection of choroidal melanoma via tear-derived protein nanoparticles	Tear samples (18 = uveal melanoma, 18 controls)	Random forest, support vector machine, decision tree, deep neural network, transfer learning with CNN (VGG16, ResNet50, Xception)	Accuracy (VGG16) 98%

AI: artificial intelligence; CHRPE: congenital hypertrophy of the retinal pigment epithelium; CNN: convolutional neural network; MOLES: Mushroom shape, Orange pigment, Large size, Enlargement, Subretinal fluid; MRI: magnetic resonance imaging; OCT: optical coherence tomography.

**Table 7:** Summary of AI research in management and prognostication of uveal melanoma and related lesions.

Citation	Problem/Challenge	Dataset	AI Methods	Performance Metrics
Kaiserman I et al., 2006 [77]	Predict malignant transformation in choroidal nevi	Clinical data, ultrasound parameters (*n* = 659 eyes)	Artificial neural networks	Area under curve 72%
Tailor PD et al., 2024 [78]	Predict choroidal nevus transformation to melanoma	Fundus photographs, auto fluorescence, OCT, and B-scan ultrasonography (*n* = 2870 nevi)	SAINTS and SAINTSLite (XGBoost, LGBM, Random Forest, Extra Tree)	Accuracy: SAINTS 89% SAINTSLite 91%
Taktak AF et al., 2004 [79]	Predict survival in UM	5 clinical parameters (*n* = 2331 patients)	Cox regression, artificial neural networks	Area under curve 72 to 96%
Kaiserman I et al., 2005 [80]	Prognosticate survival in UM	5 clinical parameters (*n* = 153 patients)	Artificial neural networks; Logistic regression	Accuracy: ANN 84% Logistic regression 86%
Damato B et al., 2008 [81]	Estimate survival probability in UM	Clinical, cytogenetic and histopathological parameters (*n* = 2654 patients)	Conditional hazard estimating neural network (artificial neural network)	C-index 0.8
Chen YN et al., 2022 [82]	Predict metastatic and survival outcomes in UM	Clinical parameters (*n* = 1553 patients)	Random Forest	Area under curve Death 93% Metastasis 87%
Wu SN et al., 2024 [83]	Predict distant metastasis in UM	Clinical data (4189 patients, Surveillance, Epidemiology, and End Results database)	Multilayer Perceptron, SHAP	Area under curve 88%
Lever M et al., 2024 [84]	Identify UM patients at risk for liver metastasis	Clinical, radiomic parameters, Ct scans (*n* = 101 metastatic UM patients)	Cox-LASSO regression machine learning	C-index: Time to treatment failure: 0.7 Overall survival; 0.7
Fleury E et al., 2025 [85]	Predict decision of stereotactic radiotherapy or proton therapy	Clinical features (*n* = 66 UM patients)	Random forest, Multi-output regressor	Accuracy: 77–93%
Giannuzzi F et al., 2025 [86]	Assess ChatGPT 4’s ability to provide recommendations for patients with UM	Comprehensive case descriptions (text) of patients with UM (*n* = 40)	ChatGPT 4.0	Concordance with actual management: 55% Significantly different from oncology specialists (*p* < 0.05)
Tahmasebzadeh A et al., 2025 [87]	Predict local recurrence in UM after brachytherapy	Demographics and clinical data (*n* = 167 UM patients)	Logistic regression, random forest, support vector machine, gradient boosting, AdaBoost, XGBoost	Accuracy (random forest) 82%
Tahmasebzadeh A et al., 2025 [88]	Predict tumor response patterns to brachytherapy	Ultrasonography B scan (*n* = 192 patients)	DenseNet121, ResNet34	Accuracy (DenseNet121) 80%
Sachpekidis C et al., 2026 [89]	Prognosticate the treatment response to tebentafusp	18-FDG LAFOV PET CT (*n* = 15 metastatic UM patients)	Deep-learning-based segmentation (RECOMIA platform); AI-assisted PERCIST 1.0 criteria, biomarker: TMTV total metabolic tumor volume; TLG total lesion glycolysis	Elevated TMTV/TLG associated with shorter overall survival (*p* < 0.05)
Akram F et al., 2024 [90]	Predict molecular subclass of UM	Whole slide images (*n* = 113 patients)	EfficientNet, Vision Transformer, Swin Transformer	Accuracy 75%
Chen H et al., 2026 [91]	Automate cancer subtyping from digital cytopathology images	Digital cytopathology images (100 samples from 88 UM patients)	YOLACT (Instance segmentation), UMAP, explainable Bayesian rule set	Accuracy 88% (UM gene expression profile class prediction)
Wan Q et al., 2025 [92]	Identify high risk immune infiltration profiles	Whole slide images (138 UM patients)	CellProfiler software; Inception-V3, SVM, Random Forest	Area under curve (nomogram): 90%
Liu TYA et al., 2020 [93]	Gene expression profile prediction in UM	Cytopathology slides (*n* = 20 slides)	Transfer learning, ResNet-152	Accuracy 75%
Khorshid Sokhangouy S et al., 2025 [94]	Identify a prognostic signature in UM	Bioinformatics data (miRNA, *n* = 80 patients)	Univariate Cox proportional hazards regression, Multilayer perceptron	Area under curve (10 miRNA) 70%
Sun Y et al., 2024 [95]	Identify macrophage-related genes in primary and metastatic UM	UM single-cell dataset GSE139829 (web)	Single-cell RNA-seq High-dimensional weighted gene co-expression network analysis (hdWGCNA) and deep learning (CNN, SVM, LASSO)	Area under curve 81% (primary vs. metastasis)
Liu J et al., 2023 [96]	Construct glycosylation gene signature to prognosticate UM	Clinical data, gene expression profile (*n* = 80 TCGA cohort, 28 GSE84976 cohort)	LASSO Cox regression	Area under curve 89%
Chi H et al., 2022 [97]	Construct sphingolipid metabolism gene signature to prognosticate UM	Clinical data, gene expression profile (*n* = 80 TCGA cohort, 28 GSE84976 cohort)	LASSO-Cox regression, Support vector machine recursive feature elimination	Area under curve 93%
Donizy P et al., 2022 [98]	Compare clinicopathological variable to gene expression signatures for prognostication of UM	Clinical parameters, pathological features, gene expression profiles (*n* = 164 patients; and 80 from TCGA cohort)	Cox proportional hazards, random survival forest, survival gradient boosting	Area under curve Overall survival 73% Progression-free survival 77%
Hou P et al., 2021 [99]	Identify prognostic signature for UM	Methylome, transcriptome (80 TCGA; 57 GSE44295; available online)	CoxBoost (Likelihood-based boosting)	Area under curve 95%
Zhang Z et al., 2020 [100]	Create an immune-related risk signature for UM	mRNA profile and clinical records (*n* = 80 UM patients, from online database)	Cox-LASSO regression	Area under curve 95%

AI: artificial intelligence; ANN: artificial neural networks; CNN: convolutional neural network; FDG LAFOV PET CT: Fluorodeoxyglucose Long-Axial Field-of-View Positron Emission Tomography; LASSO: least absolute shrinkage and selection operator; LGBM: Light Gradient Boosting Machine; OCT: optical coherence tomography; SHAP: SHapley Additive explanation; SVM: Support Vector Machine; TCGA: The Cancer Genome Atlas; UM: Uveal melanoma; UMAP: Uniform Manifold Approximation and Projection; YOLACT: You Only Look At CoefficienTs.

**Table 8:** Summary of AI research in other intraocular tumors.

Citation	Problem/Challenge	Dataset	AI Methods	Performance Metrics
Zou J et al., 2024 [101]	Prediction of ocular metastasis from gastric carcinoma	Clinical and laboratory data of 3532 patients	Gradient boost model, SHAP method	Accuracy 99%
Sun JQ et al., 2023 [102]	Clinical predictive model for ocular metastasis in liver cancer	Clinical and laboratory data of 1540 patients	Extreme gradient boost machine learning model	Accuracy 99%
Kuo DE et al., 2017 [103]	Developing a model to distinguish between endophthalmitis vs. uveitis vs. lymphoma	Aqueous and vitreous interleukin levels (7 = endophthalmitis, 29 = lymphoma, 49 = uveitis)	Gradient-boosted decision tree classifier	Accuracy 97%
Li S et al., 2025 [104]	Developing a model to diagnose primary vitreoretinal lymphoma through complete blood count screening	Complete blood count data (*n* = 255 patients, *n* = 292 controls)	Random forest model, SHAP method	Sensitivity 95% (validated in a community cohort of 100,526 patients)
Nezu N et al., 2021 [105]	Predicting ocular diagnosis in various scenarios	Immune mediators in aqueous humor (512 eyes with 17 intraocular disease)	Random forest, linear SVM, radial basis function SVM, decision tree, and naïve Bayes classifier models	Accuracy > 90%
Sensoy E et al., 2023 [106]	Assessing the response of AI chatbots to questions on intraocular tumors	36 questions from American Academy and Ophthalmology 2022–2023 Basic and Clinical Science Course Ophthalmic Pathology and Intraocular Tumors book	Bing, Bard, ChatGPT AI chatbots	Correct answers: Bing 64% Bard 69% ChatGPT 53%
Sun J et al., 2025 [107]	Development of a comprehensive dataset for development and validation of AI models	Fundus images (*n* = 2031)	ResNet50, ResNet101, ConvNeXt-T, ViT-B models	Accuracy (Vit-B) 79%
Fleury E et al., 2024 [108]	Development of a model for monitor units determination in proton therapy	Treatment doses of 3415 patients	Decision tree, random forest, extra trees, K-nearest neighbors, gradient boosting, and the support vector regression.	Predicted output within 10% for 99% of the patients
Goswami M et al., 2021 [109]	Differentiating tumor region and perform segmentation of tumor volumes by deep learning	OCT and OCT angiography of 50 mice eyes	SVM, URsD, URsEn, Ulncp, UVgg models	Dice coefficient 85%
Nagarajan A et al., 2025 [110]	Automating tumor localization and segmentation of retinal tumors in OCT images	OCT images (*n* = 3815)	Dependent Inter-Feature Segmentation Method	Dice coefficient 96%

OCT: optical coherence tomography; SVM: Support Vector Machine.

## Data Availability

No new data were created or analyzed in this study.

## Supplementary Materials

The following supporting information can be downloaded at: https://www.mdpi.com/article/10.3390/cancers18111698/s1, Table S1: List of articles included in the review and overview of domains; Table S2: Assessment of artificial intelligence technologies; File S1: PRISMA checklist.

## Abbreviations

The following abbreviations are used in this manuscript:

AI	Artificial intelligence
ANN	Artificial neural network
ASOCT	Anterior segment optical coherence tomography
CHRPE	Congenital hypertrophy of retinal pigment epithelium
CNN	Convolutional neural network
CT	Computed tomography
FDG	Fluorodeoxyglucose
LAFOV	Long-Axial Field-of-View
LASSO	Least absolute shrinkage and selection operator
LGBM	Light Gradient Boosting Machine
LIME	Local Interpretable Model-Agnostic Explanations
MOLES	Mushroom shape, Orange pigment, Large size, Enlargement, Subretinal fluid
MRI	Magnetic resonance imaging
OCT	Optical coherence tomography
OSSN	Ocular surface sqamous neoplasia
PET	Positron Emission Tomography
RB	Retinoblastoma
SHAP	SHapley Additive explanation
SVM	Support Vector Machine
TCGA	The Cancer Genome Atlas
UM	Uveal melanoma
UMAP	Uniform Manifold Approximation and Projection
YOLACT	You Only Look At CoefficienTs
YOLO	You Only Look Once
